# Odontostomatologic management of patients receiving oral anticoagulant therapy: a retrospective multicentric study

**DOI:** 10.1186/1750-1164-5-5

**Published:** 2011-07-19

**Authors:** Francesco Inchingolo, Marco Tatullo, Fabio M Abenavoli, Massimo Marrelli, Alessio D Inchingolo, Salvatore Scacco, Francesco Papa, Angelo M Inchingolo, Gianna Dipalma

**Affiliations:** 1 Department of Dental Sciences and Surgery, University of Bari, Bari, Italy; 2 Department of Medical Biochemistry, Medical Biology and Physics, University of Bari, Bari, Italy; 3 Department of "Head and Neck Diseases", Hospital "Fatebenefratelli", Rome, Italy; 4 Department of Maxillofacial Surgery, Calabrodental, Crotone, Italy; 5 Department of Dental Sciences and Surgery, Dental School, Bari, Italy; 6 Department of Surgical, Reconstructive and Diagnostic Sciences, University of Milano, Milano, Italy

**Keywords:** Oral Anticoagulant Therapy (OAT), Tranexamic Acid, Oral Surgery

## Abstract

**Introduction:**

Today, we frequently find patients taking oral anticoagulant therapy (OAT), a prophylaxis against the occurrence of thromboembolic events. An oral surgeon needs to know how to better manage such patients, in order to avoid *hemorrhagic *and *thromboembolic *complications.

**Materials and methods:**

A group of 193 patients (119 men aged between 46 and 82 and 74 women aged between 54 and 76) undergoing OAT for more than 5 years were managed with a standardized management protocol and a 2-months follow-up. The aim of the present study was to apply a protocol, which could provide a safe intra- and postoperative management of patients on OAT.

**Results:**

Among the 193 patients, only 2 had postoperative complications.

**Conclusions:**

We think that the protocol used in the present study can be used for complete safety in the treatment of this type of patients.

## Introduction

The typology of coagulation disorders is extremely heterogeneous. Today, we frequently treat patients taking oral anticoagulant therapy (OAT), a prophylaxis against the occurrence of thromboembolic events [1,2].

Recent studies on the Italian population indicate an estimated 250.000 patients taking OAT to date. Indications for use of this pharmacological prophylaxis [3,4] are confirmed by cardiac diseases, vasculopathies with thromboembolic etiology, a few enzymatic-receptorial anomalies, as well as qualitative/quantitative alterations of some cellular hematic components [5,6].

Today, we deal with patients undergoing dicoumarolics therapy, which interfere with the metabolism of vitamin K and therefore with the synthesis of coagulation factors-- II, VII, IX, and X [7,8]. These patients often take the most frequent OAT, such as:

• **acenocoumarol**: intermediate action, which begins within 24-48 h and ends within 2-4 days.

• **warfarin**: long and delayed action from 3 to 7 days.

Warfarin has a more permanent and long-lasting effect, because of its longer biological half-life (about 36 h).

The aim of the present study was to apply a protocol, which could provide a safe intra- and postoperative management of patients on OAT.

## Materials and methods

In the present multicentric study, we focused on a cohort of 193 patients (119 men aged between 46 and 82 years and 74 women aged between 54 and 76 years) undergoing OAT for more than 5 years.

This study is carried out in compliance with the Helsinki Declaration; it is divided into a *preoperative phase*, an *intraoperative phase*, and a *postoperative *phase including a 2-months follow-up.

### a) Preoperative Phase

Medical history was carried out through specific questionnaires for the diagnosis of hemorrhagic disorders [9]. We asked the patients if they ever had hemorrhagic diathesis, its type (spontaneous or post-traumatic/post-surgical), and outcome. It is important to ask the patients if they drink alcohol or take unnecessary substances [10]. A careful assessment of the ongoing pharmacological therapy should be carried out. Some currently used drugs (such as penicillin and cephalosporin, trimetoprim, gentamicin, rifampicin, analgesics and non-steroidal anti-inflammatory drugs (NSAIDS), cardiovascular and anti-diabetic drugs, diuretics, platelet anti-aggregants, heparins, gold salts, and anti H_2_) can produce thrombocytopenia [5].

• On the day of the surgery, the dental surgeon has to investigate the patient's hemocoagulative structure through a series of hematoclinical examinations.

Counseling between the dental surgeon, patient, and hygienist is necessary to make patients aware of the importance of dental hygiene in preventing dental and periodontal pathologies, which could later require more destructive and invasive therapies [11].

In addition to this, inflammatory or degenerative pathologies of the oral cavity play a crucial role in the genesis of bacterial endocarditis, a non-rare phenomenon among chronic valvulopathy patients and among those with valvular prostheses [12].

Bacterial Endocarditis (BE) is a potentially lethal disease, even if patients are on antibiotics. Mortality can reach up to 30% of cases.

The *British Society Antimicrobial Chemotherapy *(BSAC) has revised the cardiac table of patients risking BE. According to these new indications, there are 3 types of patients risking BE:

1) patients with previous BE; 2) patients with cardiac valve replacement; and 3) patients who underwent surgical shunt creation, especially in the cardiopulmonary area.

These new guidelines are no longer ambiguous about performing an antibiotic prophylaxis (table 1) apart from the routinely removal of infections during the days preceding the oral surgery.

**Table 1:** Pattern of Antibiotic Prophylaxis

DRUGS	DOSAGE
**FIRST CHOICE**	

***Amoxicillin***	Adults: 2 gm per os, 1 hour before surgery 1,5 gm per os, 6 hours after initial dose Children: 50 mg/Kg per os, 1 hour before surgery

**PATIENTS UNABLE TO TAKE DRUGS PER OS**	

***Ampicillin***	Adults: 2 gm i.m., before surgery Children: 50 mg/Kg ev or im, 30 min before surgery

**IN CASE OF ALLERGY TO PENICILLIN**	

***Clindamycin*** Cephalexin* or Cephadroxil* Azithromycin or Clarithromycin	Adults: 600 mg per os, 1 hour before surgery Children: 20 mg/Kg per os, 1 hour before surgery Adults: 2 gr per os, 1 hour before surgery Children: 50 mg/Kg per os, 1 hour before surgery Adults: 500 mg × os 1 hour before surgery Children: 15 mg/Kg per os, 1 hour before surgery

**ALLERGIC PATIENTS UNABLE TO TAKE DRUGS PER OS**	

***Clindamycin*** Cephazolin*	Adults: 600 mg ev, 30 min before surgery Children: 20 mg/Kg ev, 30 min before surgery Adults: 1 gr im, 30 min before surgery Children: 25 mg/Kg im or ev, 30 min before surgery

*provided that there are no serious allergic reactions to penicillin

The aim of antibiotic prophylaxis is to have an effective serum concentration of the active principle during the whole perioperative period.

The antibiotic therapy should be given shortly after the beginning of the treatment (1-2 h) and within 6-8 h from surgery; a longer therapy is justified only in case of infected tissues or delayed wound healing.

### b) Intra-operative phase

Fifteen days before surgery, periodontal preparation of patients through tartar ablation by means of an ultrasonic equipment. To avoid periodontal hemorrhages, it is necessary to invite patients (before ablation) to do oral rinses for 1 min, with 10 ml of 4.8% solution of tranexamic acid. Moreover, it is important to prescribe to patients oral rinses with *chlorhexidine digluconate *0.12%, 2 times a day for 14 days before surgery.

The day of surgery, it is necessary to assess the hemocoagulative parameters by taking a blood sample, which will allow to evaluate PT, PTT, and especially the INR measured 24 h before surgery.

In the last 3 years, we treated 193 patients on OAT with a standardized management and a 2-months follow-up. Although we evaluated every single clinical case in its uniqueness, we wanted to apply a protocol providing for a safe intra- and postoperative management of patients receiving OAT.

There are not any contraindications to the use of local anesthetics: mepivacaine, lidocaine, or articaine are recommended (articaine is needed when an intense ischemia is required and when surgery lasts more than 1 h, thanks to its longer action than mepivacaine and to its good effectiveness in controlling postoperative pain).

The literature does not recommend the use of vasoconstrictors as they could cause tachycardia episodes [13]; nevertheless, we think that a safe use of a vasoconstrictor is possible by means of an aspirating syringe (*carpule) *and a good anesthesia technique, except for patients who recently had a myocardial infarction or coronary artery bypass surgery (≤ 8 months).

This will bring 2 advantages:

**1) **The vasoconstrictor will guarantee an optimal zonal ischemia and a longer anesthetic effect, which is very useful when treating hemorrhagic patients [14];

**2) **The vasoconstrictor (*red *phials) allows for an effective anesthesia, using a smaller quantity of anesthetic compared to *green *phials (anesthetic without vasoconstrictor).

Among the hemostatic agents that a dental surgeon can use, there are ferric sulfate, oxidized cellulose, gelatin sponges, and anti-fibrinolytics.

Oxidized regenerated cellulose [15] (*Surgicel, Tabotamp*) consists of a sterile gauze and oxidized cellulose. First, it acts as a mechanical barrier and then as a viscous mass, which works as an artificial clot.

Antifibrinolytics (*Tranex, Ugurol*) perform a good local hemostatic action.

Local use of tranexamic acid to prevent and treat hemorrhage in the oral cavity is recommended. The use of this active principle encourages clot formation and reduces the bleeding time [16].

Before surgery, we perform a standardized preoperative procedure.

We recommend a pre-anesthesia by administering benzodiazepines (15 gtts, 20 min. before surgery). As a matter of fact, premedication allows for greater patient compliance during surgery, and the adrenalin plateau caused by intraoperative stress will be avoided.

We suggest to our patients oral rinses with chlorhexidine digluconate 0.2% solution [17].

A careful disinfection of perioral and intraoral mucus-dental tissues by using an iodized solution is also necessary. Rinsing the operating field with tranexamic acid solution (4.8%) allows to control hemostasis since initial dieresis.

Performing a venous access allows to operate quickly in case of hemorrhagic emergency. In this case, patients could have hypotensive phenomena, which involve difficult identification of the venous access, because vessels could be partially collapsed.

In major oral surgery, 2 g of tranexamic acid are administered intravenously (IV) 1 h before surgery (divided into 4 × 0.5 g doses every 15 min) and after preparing the venous access [16].

The cardiac activity and the pressure status have to be steadily monitored using specific equipment (CardiocapII, Datex).

The surgical procedure is performed following a standardized controlled protocol.

A *plexus, intraligamentous*, or *locoregional *anesthesia is performed using a slow infusion of mepivacaine 3%; we have described above what we think about the use of vasoconstrictors, which will bring more benefits than objective risks. Following a periotome syndesmotomy, a luxation of the dental-alveolar gomphosis will be performed. We suggest to reduce to a minimum the use of surgical elevators, because the moments of force of these instruments cause severe trauma to soft tissues along with microlesions to the alveolar ridge. After avulsion, manual reduction of the "greenstick" fracture [2] of the post-extractive alveolus will be performed by two-finger compression of the vestibular and lingual-palatal alveolus portions, in order to reduce the fracture occurred during avulsion.

Accurate alveolar bone cleaning is to perform: by means of an alveolar spoon, we will remove all the possible granulation tissues representing the *primum movens *in the residual cyst formation, namely post-extractive alveolites [1,2].

A bone rongeur or a bone mill with a turbine motor (continuous irrigation) allows to eliminate the bone roughness, which could damage the soft tissues of the antagonist arch. We compressed the post-extraction alveolus with oxidized regenerated cellulose.

we suggest an *absorbable *suture (*Vicryl 3/0 *or *4/0 *to avoid trauma of suture removal) using a "simple running stitch," with a further *safety *suture using a "simple interrupted stitch"; then, there will be a 30 minute compression of the surgical area, followed by sterile gauze application filled with tranexamic acid. If bleeding stops about 30 min later, it is the clinical demonstration of the technical success of surgery.

### c) Post-operative phase

Patients should be discharged at least 60 minutes after surgery (in order to verify the absence of hemorrhagy); we advise a cold and liquid diet during the next 3 days and a tepid and semiliquid diet during the following 3 days.

Patients should perform oral rinses for 2 minutes with 10 ml of tranexamic acid, 4 times a day for 7 days [16]. After rinsing, patients should not drink or eat for about an hour.

During the first 30 days after surgery, we suggest patients to rinse their mouth every 12 h with chlorhexidine digluconate 0.12% solution; anti-inflammatory therapy should be prescribed only in case of complex surgeries or if patients experience postoperative pain: 500 mg of paracetamol every 8 hours will be a precaution against hemorrhagic problems caused by the NSAIDs.

The highest risk of postoperative hemorrhagic diathesis is during the first 12 h after the surgery; our follow-ups allow us to exclude hemorrhagic relapse from the 6^th ^day after the surgery.

In case of postoperative hemorrhage, a buffer imbibed in tranexamic acid should be applied together with compression of the area [18]; if bleeding persists, up to 2 g of tranexamic acid divided into 4 doses of 0.5 g has to be administered. In incoercible cases, it is indispensable to consult the hematologist in order to reduce OAT and develop a strategy to stop hemorrhagic diathesis [19,20].

We recommend the use of absorbable sutures (*Vicryl 3/0 or 4/0) *to avoid trauma caused by suture removal. Non-absorbable sutures are particularly indicated for areas presenting aponeurosis, mimic muscles, frenula, etc. Removal should nonetheless be performed on the 8^th ^day and in the less traumatic way. In case of slight hemorrhages, it is enough to apply a tampon of tranexamic acid and repeat oral rinses for 1 or 2 days.

Regular follow-ups should be planned (7, 15, 30, and 60 days after surgery). They include intra- and extraoral examinations, a measurement of symptoms of pain by visual analogue scale (VAS), the motivation of patients to oral hygiene, and the possibility of further dental treatments.

## Results

Out of 193 treated patients, just 2 of them (only 1.03% of cases) had problems related to surgery: the 1^st ^one had a slight hematoma in the area of labial commissure, due to the trauma caused by the Farabeuf retractor, but it regressed in a few days. The 2^nd ^one had pains and did not present clot formation in the post-extractive site. Intraoral examination confirmed the diagnosis of post-extractive alveolitis, while the forthcoming pathological medical history indicated that the patient did not follow hygiene rules (smoker) in the immediate postoperative period; the alveolus was cleaned and the symptoms significantly reduced in the follow-up period.

The results of this study show a protocol which allows a safe treatment of patients on OAT.

The problems that may principally arise in this type of surgical patients are related to a deficient or excessive clotting, we may also have problems of excessive susceptibility to bacterial infections. With this protocol we have treated 193 patients and we have had none of these problems, except in only 2 cases. These data are significant and show that the management of these patients is possible if we follow a proper protocol such as this one here described.

## Discussion

Dental procedures represent a particularly common intervention for patients receiving anticoagulant therapy. The most recent literature indicated that in most cases, no change in the intensity of anticoagulation is needed. As we can highlight in the literature, there are no well-documented cases of severe bleeding in this setting, but there are many documented cases of embolic events in patients whose warfarin therapy was discontinued for dental treatment. If there is a need to control local bleeding, tranexamic acid administration has been used successfully without interrupting anticoagulant therapy [18,19].

The diathesis of bleeding is significantly related to the intensity of the anticoagulant effect. Therefore, in patients who show a severe bleeding, every effort should be made to maintain the INR at the lower limit of the therapeutic range. For patients with mechanical prosthetic valves (and a persisting risk of increased bleeding), it would be reasonable to aim for an INR range of 2.0 to 2.5. For patients with atrial fibrillation (and a persisting risk of increased bleeding), the anticoagulant intensity can be reduced to an INR range of 1.5 to 2.0 with the expectation that efficacy will be reduced but not abolished. Alternatively, aspirin can be used to replace warfarin in patients with atrial fibrillation, but also with a reduced efficacy in high-risk patients [21].

In the past, the scientific literature often recommended OAT interruption in order to avoid intra- and postoperative hemorrhagic risk: in a research carried out in 1996 in the USA, around 70% of clinicians recommended their patients to interrupt anticoagulant treatment before undergoing dental treatments, with a risk of hemorrhage. In this light, we want to underline how the interruption of an anticoagulant therapy, even for a short time, exposes the patient to thromboembolic risks, even many days after treatment: the analysis of 500 documented cases of patients who suspended an anticoagulant therapy to undergo oral surgery revealed that 5 patients (0.95%) had thromboembolic episodes; 4 of them had fatal thromboembolism, while 1 patient had 2 embolic non-fatal episodes [3].

A randomized study showed how OAT interruption to perform minor oral surgery does not imply any real clinical benefit [4,5].

Even in those cases in which we are performing surgical treatments particularly invasive, or we are treating a syndromic condition closely related to an augmented bleeding risk, we can emphasize the effectiveness of the association between a conscious administration of tranexamic acid and a well done surgical performance, especially if we follow a well documented protocol [22,23].

## Conclusions

We think that the protocol analyzed in the present study can be used in complete safety for the treatment patients receiving OAT, even though a few factors can alter the patient response to the protocol. The recommendations that urge to maintain the INR at the lower limit of the therapeutic range are still actual, although, the evaluations to do are closely related to each patient, to his general conditions and to the typology of oral surgery to perform.

## Consent

Written informed consent was obtained from the patients for publication of this case report and accompanying images. A copy of the written consent is available for review by the Editor-in-Chief of this journal.

## Competing interests

The authors declare that they have no competing interests.

## Authors' contributions

FI and FMA participated in the surgical treatment and in the follow-up examinations. MT and SS drafted the manuscript and revised the literature sources. MM and GD participated in the follow-up examinations. FP analyzed the data carried out from the multicentric study and revised the literature sources.

ADI revised the literature sources. AMI managed the data collection and contributed to writing the paper. All authors read and approved the final manuscript.
